# Real-life data on the efficacy and safety of erenumab in the Abruzzo region, central Italy

**DOI:** 10.1186/s10194-020-01102-9

**Published:** 2020-04-07

**Authors:** Raffaele Ornello, Alfonsina Casalena, Ilaria Frattale, Amleto Gabriele, Giannapia Affaitati, Maria Adele Giamberardino, Maurizio Assetta, Maurizio Maddestra, Fabio Marzoli, Stefano Viola, Davide Cerone, Carmine Marini, Francesca Pistoia, Simona Sacco

**Affiliations:** 1 Neuroscience Section, Department of Applied Clinical Sciences and Biotechnology, University of L’Aquila, L’Aquila, Italy; 2 Department of Neurology, ‘G. Mazzini’ Hospital, Teramo, Italy; 3 Neurology Service, ‘SS. Annunziata’ Hospital, Sulmona, Italy; 4 Department of Medicine and Science of Aging, ‘G. D’Annunzio’ University, Chieti, Italy; 5 Department of Neurology, ‘F. Renzetti’ Hospital, Lanciano, Italy; 6 Department of Neurology, ‘S. Pio da Pietrelcina’ Hospital, Vasto, Italy; 7 Department of Neurology, ‘S. Salvatore’ Hospital, L’Aquila, Italy; 8 Department of Life, Health and Environmental Sciences, University of L’Aquila, L’Aquila, Italy

**Keywords:** Migraine, Calcitonin gene-related peptide, Migraine prevention, Monoclonal antibodies, Erenumab, Real-life study

## Abstract

**Background:**

We aimed to assess the efficacy and safety of erenumab, a fully human monoclonal antibody inhibiting the calcitonin gene-related peptide receptor (CGRPr), for the prevention of migraine in a real-life setting.

**Main body:**

We included in our observational study all patients with episodic or chronic migraine treated with erenumab during the year 2019 in the Abruzzo region, central Italy, and with a 6-month follow-up. We included 89 patients; 76 (85.4%) received 6 doses of erenumab, 11 (12.4%) autonomously withdrew the drug due to perceived inefficacy, and 2 (2.2%) due to adverse events. Seventy-eight patients (87.6%) were female, with a mean age of 46.8 ± 11.2 years; 84 (94.4%) had chronic migraine, and 64 (71.9%) medication overuse. All patients had ≥2 prior preventive treatment failures. Fifty-three patients (69.7%) had a 50% decrease in monthly migraine days (MMDs) within the first three doses; 46 (71.9%) of 64 patients withdrew medication overuse. In the 76 patients who completed a 6-dose treatment, erenumab decreased median MMDs from 19 (interquartile range [IQR] 12–27.5) to 4 (IQR 2–9.5; *P* < 0.001), median monthly days of analgesic use from 10 (IQR 4.5–20) to 2 IQR 0–5; P < 0.001), and median monthly days of triptan use from 5 (IQR 0–15.5) to 1 (IQR 0–4; P < 0.001). We recorded 27 adverse events in 20 (22.5%) patients, the most common being constipation (13.5%). One adverse event, i.e. allergic reaction, led to treatment discontinuation in one patient.

**Conclusions:**

Our real-life data confirm the efficacy and tolerability of erenumab for the prevention of migraine in a difficult-to-treat population of patients with a high prevalence of chronic migraine and medication overuse.

## Background

Migraine is the third most prevalent and the second most disabling disease worldwide [1]. Migraine can be classified as episodic (EM) or chronic (CM) according to the number of monthly headache days [2]. According to the World Health Organization (WHO), migraine ranks third among the most disabling conditions of the human kind [3] and is the first cause of disability under 50 years of age [4]. Despite its significant burden, the available preventive treatments for migraine are not specific [5, 6] and poorly tolerable [7, 8], while botulinum toxin A is effective only on CM [9, 10]. Given this background, the advent of monoclonal antibodies against the calcitonin gene-related peptide (CGRP) or its receptor (CGRPr) represent a breakthrough in migraine prevention [11–13]. Evidence from randomized controlled trials strongly supports the efficacy and safety of those agents in the prevention of both EM and CM [14, 15]. However, real-life data are needed to confirm the results of clinical trials, provide evidence to meet the needs of common clinical practice, and possibly improve the treatment protocols.

Erenumab, a fully human monoclonal antibody directed against CGRPr, is the first approved migraine-specific treatment [16], whose efficacy and safety were proven in both EM and CM [17–19]. Erenumab is available in two monthly dosages, namely 70 mg and 140 mg [16], with a slight numerical advantage of the higher over the lower dosage in terms of efficacy [20]. Compared with the trials, two real-life data from the USA [21] and Italy [22] confirmed the efficacy of erenumab in EM and CM after 2 months, with a higher incidence of constipation that did not lead to drug withdrawal [21]. However, there are currently no real-life studies assessing the efficacy and safety of erenumab over more than 2 months of treatment.

In the present real-life, multicenter study, we aimed to retrospectively review the efficacy and safety of erenumab in patients with EM and CM.

## Methods

### Study population

Our study included patients aged 18 to 65 years consecutively treated with erenumab in the Headache Centers of Avezzano, L’Aquila, Sulmona, Teramo, Chieti, Lanciano, and Vasto, all located in the Abruzzo region, central Italy, from January to December 2019. The Abruzzo region hosts a population of 1,311,580 inhabitants according to the most recent data [23]. The Headache Centers of Avezzano-L’Aquila, Teramo, and Chieti offer a level 3 care, while the other offer a level 2 care according to the European Headache Federation/ Lifting the Burden (EHF/LTB) proposed classification [24]. The study was approved by the Internal review Board of the University of L’Aquila with the number 44/2019. All patients signed an informed consent.

The Avezzano-L’Aquila center started treating patients with erenumab in January 2019 and therefore recruited more patients than the remaining centers, which started adopting erenumab treatment from April 2019 onwards. In the absence of established reimbursement criteria from the Italian Agency for Drug administration (AIFA), erenumab was provided to patients from the producing company upon reasonable request from the Headache Centers. The drug was provided for patients with migraine with or without aura diagnosed by expert physicians according to the International Classification of Headache Disorders (ICHD) criteria [2]. All the study patients had to have > 4 monthly migraine days and failure of ≥2 prior preventive treatments for migraine, according to the eligibility criteria established by the European Headache Federation [14] and the American Headache Society [25]. Due to the limited availability of the drug, the study centers focused on the most difficult-to-treat patients, i.e. those with a long history of disabling migraine and/or previous treatment failure. Following the exclusion criteria of the available trials [17–19, 26], we excluded from treatment patients with major medical or psychiatric illnesses.

### Treatment procedure

Erenumab was administered during in-person visits in a monthly subcutaneous dose of 70 mg, with the option of switching to 140 mg monthly (i.e., two 70 mg doses) in case of a < 30% decrease in monthly migraine days (MMDs) compared with baseline; the dose escalation could be done since Dose 2 or even at treatment start in patients with several prior preventive treatment failures, according to the results of the LIBERTY trial [19]. In all patients, erenumab treatment was intended to be continued at least until Dose 6, but we acknowledged the possibility of early withdrawal because of severe adverse events, lack of compliance, or ineffectiveness (< 30% reduction in MMDs and/or lack of satisfaction with treatment).

Patients were allowed to start or continue concurrent oral preventive treatments for migraine at physicians’ discretion while the concurrent administration of botulinum toxin A for CM was not allowed. Withdrawing or adding concurrent oral preventive treatments for migraine was allowed over the course of treatment according to physicians’ judgement. Patients with CM and medication overuse were not detoxified prior to erenumab treatment, according to current recommendations [14].

### Data collection

For each included patient, we recorded sex, age, current treatments, and comorbidities; we also recorded age at migraine onset, age at CM onset in patients with CM, migraine frequency and intensity, associated symptoms, acute and preventive treatments as reported in the patients’ headache diaries. In each study Center, patients were asked to differentiate between migraine days, with attacks fulfilling the ICHD-3 criteria for migraine [2], and non-migraine headache days, which were not considered in the present study. We assessed attack severity by the 0–10 Numerical Rating Scale (NRS), disability by the Migraine Impact and Disability Assessment Scale (MIDAS), impact by the Headache Impact Test, 6th edition (HIT-6), and allodynia by the Allodynia Symptom Checklist-12 (ASC-12); we also recorded the scores of scales for psychiatric symptoms, including the Beck Depression Inventory (BDI) and Generalized Anxiety Disorder (GAD-7) Questionnaire. Migraine characteristics and the scores of HIT-6, NRS, and ASC-12 were assessed monthly, while the scores of MIDAS, BDI, and GAD-7 were assessed quarterly. Data were collected with a clinical interview and then reported on a standardized form with pre-determined answers which was the same for all participating centers. All the recorded data were stored in an anonymized computerized database.

### Statistical analysis

Baseline was defined as the monthly mean of the 3 months preceding erenumab treatment. Patients reporting a ≥ 50% reduction of MMDs compared with baseline to at least one dose were defined as ‘anytime responders’. The co-primary efficacy outcomes of our analyses included the decrease in monthly migraine days (MMDs), days of analgesic and triptan use, and the proportion of anytime 50% responders. Secondary efficacy outcomes included the proportions of dose-specific 50%, 75%, and 100% responders (defined according to the percent decrease in MMDs after each dose) and the decrease in mean MIDAS, HIT-6, BDI, GAD-7, and NRS scores from baseline to the month following Dose 6. The safety outcomes included adverse events, and especially serious adverse events, i.e. those leading to hospitalization, death, or treatment withdrawal. All outcome variables were assessed through headache diaries. The proportions of responders were calculated over the total of patients with complete follow-up, irrespective of treatment discontinuation, while the decrease in MMDs and days of analgesic and triptan use was calculated over the total of patients who received all the six doses.

Categorical data were reported as number and percentage, while continuous data were reported as mean ± standard deviation (SD) and scale scores were reported as median and interquartile range (IQR). We used the chi-square test to compare categorical variables and ANOVA to compare continuous variables, while we used the Mann-Whitney U test to compare medians. Statistical significance was set at *P* < 0.05. Due to the observational design of the study, we did not plan a sample size calculation.

## Results

### Patient characteristics

Among 132 patients who started erenumab treatment during the observation period, 43 (32.6%) did not yet complete a 6-month follow-up. Among the remaining 89 patients, 13 (14.6%) discontinued treatment for ineffectiveness (12 patients) or adverse events (1 patient), while the remaining 76 (85.4%) continued treatment during all the study period.

Patient characteristics are reported in Table 1. Most patients (84; 94.4%) had CM, while 5 (5.6%) had EM; 64 patients (71.9%) had medication overuse. All patients had multiple preventive treatment failures (Table 1). In detail, 53 patients (59.6%) reported failures of antiepileptics, 50 (56.2%) of antidepressants, 42 (47.2%) of botulinum toxin A, 35 (39.3%) of calcium antagonists, 22 (24.7%) of beta blockers, and 6 (6.7%) of other preventive treatments. Failures were reportedly due to ineffectiveness in 57 patients (64.1%), adverse events in 6 (6.7%), and both in 26 (29.2%).

**Table 1:** Characteristics of the study patients

Characteristics (total patients = 89)	
Female, n (%)	78 (87.6)
Age, mean ± SD	46.8 ± 11.2
Years of migraine history, mean ± SD	28.2 ± 13.3
Baseline MMDs, mean ± SD	19.8 ± 8.4
Baseline analgesic days, mean ± SD	13.5 ± 10.6
Baseline triptan days, mean ± SD	8.7 ± 10.4
Chronic migraine, n (%)	84 (94.4)
Aura, n (%)	27 (30.3)
Allodynia, n (%)	33 (37.1)
Medication overuse, n (%)	64 (71.9)
Previous preventive treatment failures, n (%)	
2	28 (31.5)
3	24 (27.0)
4	26 (29.2)
> 4	11 (12.4)
Botulinum toxin failure, n (%)	44 (49.4)
Concurrent oral preventive treatments at baseline, n (%)	37 (41.6)

**Fig. 1:**
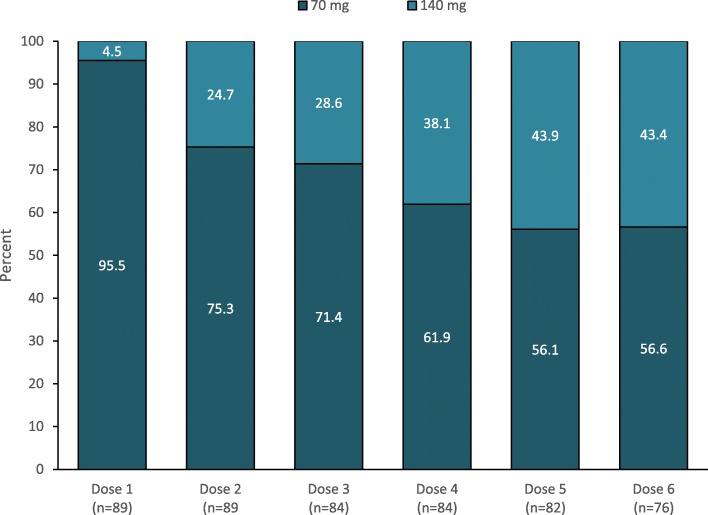
Erenumab dose escalation during the study period

In 37 (41.6%) patients, erenumab was added to an ongoing oral preventative at baseline. During the treatment period, 10 patients (11.2%) withdrew the prior oral treatment, while 8 (9.0%) started a new one. Forty-three patients (48.3%) escalated the erenumab dose from 70 to 140 mg monthly across the study period. Figure 1 reports the proportions of patients on treatment with 70 mg or 140 mg across each dose. Table 2 reports the comparison between patients escalating and not escalating erenumab dose. Patients escalating erenumab dose had a higher median number of MMDs at baseline compared with those not escalating the dose (25 vs 17; *P* = 0.002).

**Table 2:** Characteristics of patients escalating vs those not escalating the dose of erenumab during the treatment

Characteristic	Escalating (*n* = 43)	Not escalating (*n* = 46)	P value
Female, n (%)	37 (86.0)	41 (89.0)	0.753
Age, median (IQR)	50 (41–56)	47 (38–51)	0.122
Years of migraine history, median (IQR)	30 (20–36)	26.5 (18–37)	0.436
MMDs, median (IQR)	25 (17.5–30)	17 (11–25)	**0.002**
Analgesic days, median (IQR)	10 (5–28)	12 (5–19)	0.485
Triptan days, median (IQR)	6 (0–20)	0 (0–12)	0.172
Chronic migraine, n (%)	40 (93.0)	44 (95.7)	0.670
Aura, n (%)	12 (27.9)	15 (32.6)	0.651
Allodynia, n (%)	16 (37.2)	17 (37.0)	0.456
Medication overuse, n (%)	35 (81.4)	29 (63.0)	0.054
Prior preventive treatment failures, n (%)			0.501
2	15 (34.9)	13 (28.3)	
> 2	28 (65.1)	33 (71.7)	
Botulinum toxin failure, n (%)	22 (51.2)	22 (47.8)	0.753

### Responder rate, reduction in MMDs, acute medication use, attack intensity, disability, and impact

**Fig. 2:**
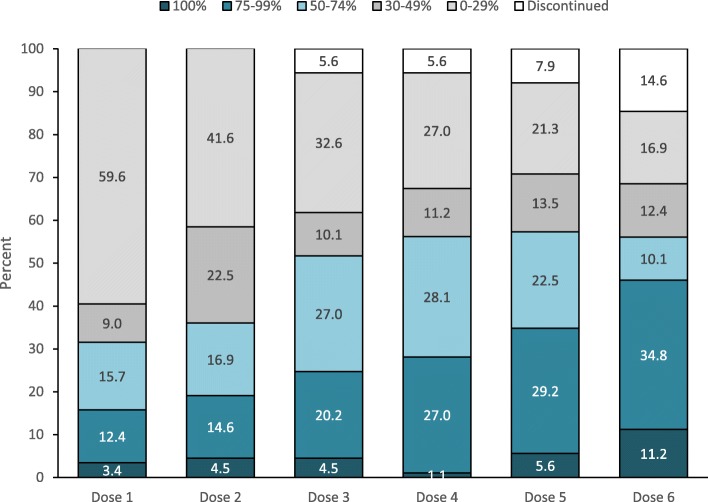
Response to each erenumab dose in the study patients

Over the study period, 64 patients (71.9%) were classified as anytime responders, i.e. had a ≥ 50% reduction of MMDs after at least one dose. Rates of dose-specific responders increased over time, ranging from 31.5% to 57.3% for ≥50% responders, from 15.8% to 46.0% for ≥75% responders, and from 1.1% to 11.2% for 100% responders (Fig. 2).

**Fig. 3:**
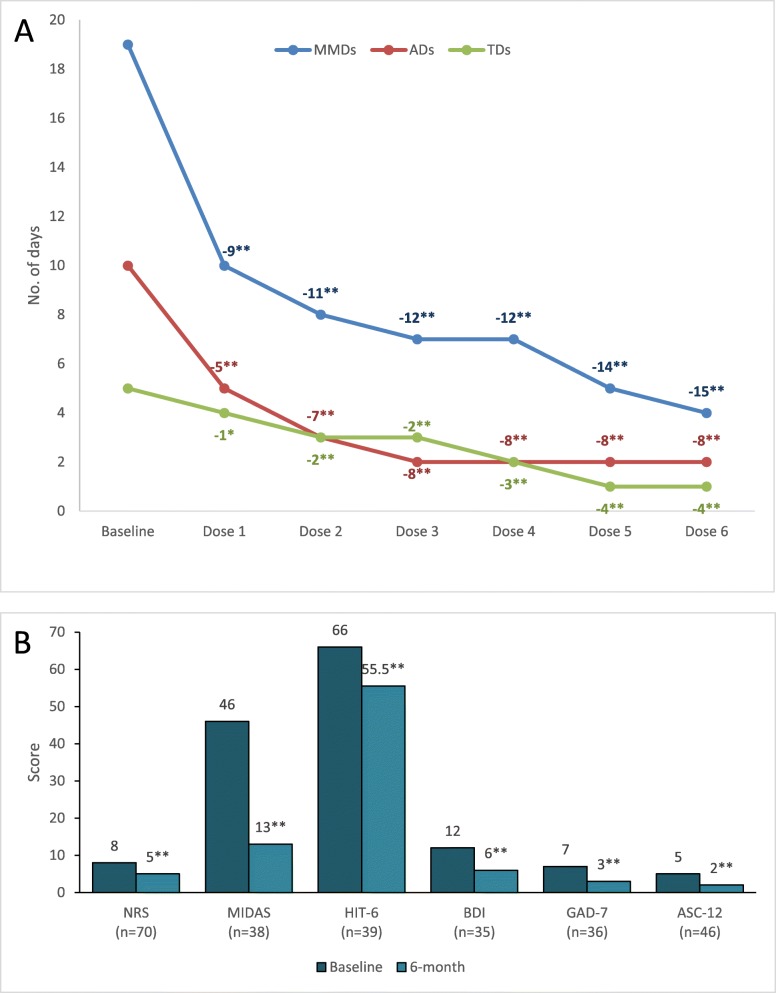
Decrease in median monthly migraine days (MMDs), analgesic use days (ADs), and triptan use days (TDs) among the 76 patients who completed treatment (panel **a**); six-month decrease in median scale scores in patients with available data (Panel **b**). All comparisons with a double asterisk have a *P* value < 0.001, while the comparison with a single asterisk has a P value = 0.001. ASC indicates Allodynia Symptom Checklist; BDI, Beck Depression Inventory; GAD-7, Generalized Anxiety Disorder Questionnaire; HIT-6, Headache Impact Test; MIDAS, Migraine Impact and Disability Assessment Score; NRS, Numerical Rating Scale

In the 76 patients who completed a 6-dose treatment, we observed a decrease in the median number of MMDs from 19 to 4 (*P* < 0.001). The mean number of analgesic use days decreased from 10 to 2 (P < 0.001), while the mean number of triptan use days decreased from 5 to 1 (P < 0.001); significant decreases of MMDs and days of analgesic or triptan use were observed since the first month of treatment (Fig. 3a). We also found that erenumab treatment improved intensity of attacks, disability, impact, allodynia as well as depressive and anxiety symptoms over the 6-month treatment period (Fig. 3b).

**Fig. 4:**
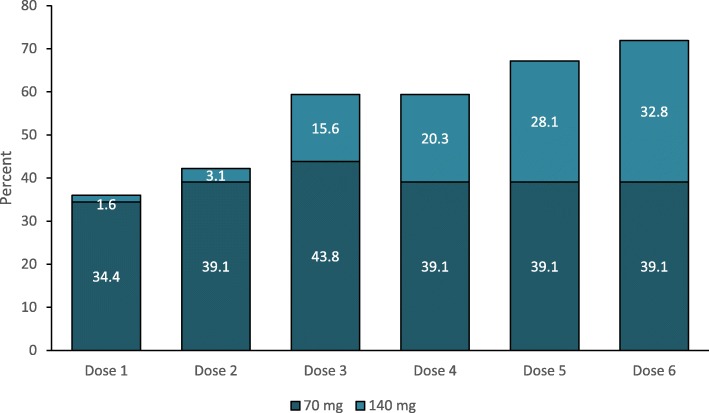
Proportion of patients withdrawing medication overuse according to erenumab dosing

Forty-six (71.9%) of the 64 patients with medication overuse withdrew the overused medication during the follow-up (Fig. 4).

### Effect of erenumab in botulinum toxin a non-responders

**Fig. 5:**
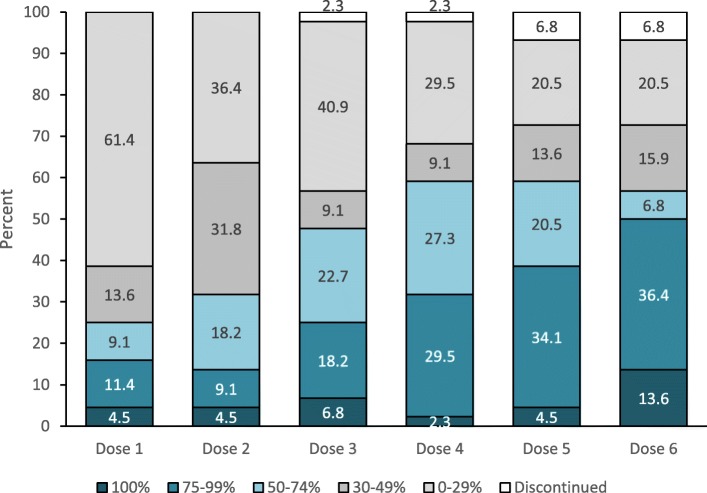
Proportion of responders among patients with chronic migraine who had failed treatment with botulinum toxin A (*n* = 44)

Among the 44 patients who had failed treatment with botulinum toxin A, 31 (70.5%) were anytime responders; the proportion of dose-specific ≥50% responders increased from 25.0% after Dose 1 to 56.8% after Dose 6 (Fig. 5).

### Onset and persistence of response

Twenty-eight patients (31.5%) had ≥50% response after Dose 1; 9 patients (10.1%) not responding to Dose 1 had ≥50% response after Dose 2, and 16 (18.0%) not responding to the first two doses had ≥50% response after Dose 3; 12 (13.5%) additional patients had ≥50% response after Dose 4 to Dose 6.

Among the 53 patients having a ≥ 50% response to at least one of the first 3 doses, 36 (67.9%) maintained the response to all subsequent doses, 5 (9.4%) to two doses, and 3 (5.7%) to one dose.

### Characteristics of non-responders

Compared with the 25 non-responders, the 64 anytime responders had a lower median number of MMDs at baseline (18 vs 26.5; *P* = 0.026) and a lower median number of monthly analgesic use days (8 vs 20; *P* = 0.008); the remaining baseline characteristics did not differ between responders and non-responders (Table 3).

**Table 3:** Comparison of the baseline characteristics of responders within the first three months of erenumab treatment versus non-responders

Characteristic	Responders (*n* = 64)	Non-responders^a^ (*n* = 25)	P value
Female, n (%)	54 (84.4)	24 (96.0)	0.134
Age, median (IQR)	48 (38–52.5)	52.5 (42–56)	0.358
Years of migraine history, median (IQR)	28 (19.5–34.5)	25 (18–32)	0.467
MMDs, median (IQR)	18 (12–27.5)	26.5 (20–30)	**0.026**
Analgesic days, median (IQR)	8 (2.5–22)	20 (11–29)	**0.008**
Triptan days, median (IQR)	3 (0–16.5)	1 (0–20)	0.532
Chronic migraine, n (%)	59 (92.2)	25 (100.0)	0.150
Aura, n (%)	18 (28.1)	9 (36.0)	0.468
Allodynia, n (%)	23 (35.9)	10 (40.0)	0.721
Medication overuse, n (%)	45 (70.3)	19 (76.0)	0.592
Prior preventive treatment failures, n (%)			0.560
2	18 (28.1)	10 (40.0)	
> 2	46 (71.9)	15 (60.0)	
Botulinum toxin failure, n (%)	31 (48.4)	13 (52.0)	0.763

^a^including 12 patients with < 50% reduction of MMDs from baseline and 13 patients who discontinued treatment

### Safety and tolerability

During the 6-month follow-up, over the 76 patients completing the 6-dose follow-up and the 13 patients who discontinued the treatment, we recorded 24 adverse events in 20 (22.5%) patients. The most common adverse event was constipation, which was observed in 12 (13.5%) patients (Table 4). One adverse event led to treatment discontinuation, namely allergic reaction (Table 4). The event resolved after treatment discontinuation.

**Table 4:** Adverse events in the study patients (*n* = 89)

Event	No. of patients (%)
Constipation	12 (13.5)
Local reaction	2 (2.2)
Pruritus	2 (2.2)
Flu-like symptoms	2 (2.2)
Abdominal cramps	1 (1.1)
Transient skin rash	1 (1.1)
Bloating	1 (1.1)
Meteorism	1 (1.1)
Nausea	1 (1.1)
Transient vaginal spotting	1 (1.1)
Urticaria^a^	1 (1.1)
Vertigo	1 (1.1)
Allergic reaction^b^	1 (1.1)
Serious adverse events	1 (1.1)
Adverse events leading to treatment discontinuation	1 (1.1)
Total	20 (22.5)

^a^Exacerbation of previous disease

^b^leading to treatment discontinuation

## Discussion

Our real-life multicenter study was performed in a difficult-to-treat population (Table 5). Notably, we treated some patients who were excluded by the trials [17–19, 26], including those with > 4 prior preventive treatment failures. Despite this, we found higher proportions of patients with 50%, 75%, or even 100% response compared with the available trials [17–19, 26], possibly due to longer treatment duration, although we cannot exclude the occurrence of placebo effect.

**Table 5:** Comparison between the randomized controlled trials of erenumab for the prevention of migraine and the present study

	ARISE [26]	NCT02066415 [17]		STRIVE [18]		LIBERTY [19]	American real-life data [21]	Italian real-life data [22]	Present study
*General characteristics*									
Migraine type	Episodic	Chronic		Episodic		Episodic	Chronic and episodic	83% chronic, 17% episodic	93.4% chronic, 6.6% episodic
Dose (mg)	70	70	140	70	140	140	70 or 140	70	70 or 140
No. of prior preventive treatment failures	< 2 (no response)	≤3 (no response)		≤2 (no response)		2–4	–	–	≤2
Follow-up duration, months	3	3		6		3	2	2	6
No. of treated patients	286	191	190	317	319	121	100	78 (13 episodic, 65 chronic)	89
*Patient characteristics*									
Female, %	85.7	87	84	84.5	85.3	80	83	75% (EM), 80% (CM)	87.6
Mean age, years	42	41.4	42.9	41.1	40.4	44.6		47.1 (EM), 47.6 (CM)	46.8
Mean migraine duration, years	22	20.7	21.9	–	–	–	–	29.1 (EM), 30.2 (CM)	28.2
Medication overuse, %	–	41	41	–	–	–	–	61.5 (EM), 84.6 (CM)	71.9
Prior preventive treatment failures, %	87.3	67	66	40.1	36.4	100	100	100	100
Mean MMDs at baseline	8.1	17.9	17.8	8.3	8.3	9.2	–	10.9 (EM), 22.0 (CM)	19.8
*Outcomes*									
MMD decrease, mean days	−2.9	−6.6	−6.6	−3.2	−3.7	−1.8	–	−7 (EM), −15 (CM)	−12.4
Triptan use days decrease, mean days	−1.2	−3.5	−4.1	−1.1	−1.6	−1.3	–	–	−5.6
50% responders, %	39.3	40	41	43.3	50.0	30	–	100 (EM), 87.5 (CM)	74.1
Adverse events %	48.1	44	47	57.3	55.5	55	34	1.3	22.5
Serious adverse events %	1.1	3	1	2.5	1.9	2	5	–	2.2

The great majority of our patients had CM, while only 5.6% had EM. Therefore, the results of our study were largely conditioned by patients with CM. Besides, patients with EM included in our study had high migraine frequency and high headache-related impact and disability, which put them close to the clinical status of patients with CM [27]. The study population likely reflects clinical practice, in which erenumab treatment is given to the most difficult-to-treat patients.

All the available randomized controlled trials of erenumab found variable 50% response rates across the different months of follow-up [17–19, 26]. In our study, we distinguished ‘anytime’ from ‘dose-specific’ responders to account for the variability of response. Although most responders had a significant response within 3 doses, new responders added after each dose of erenumab. Therefore, our data support continuing for at least 3 months and even 6 months before discontinuation. Our data also showed that response to erenumab was persistent in most cases; future studies with larger populations and longer follow-up are needed to assess the course of response to erenumab over time.

According to our data, erenumab decreased the intensity, disability and impact of headache and symptoms of depression and anxiety, which were not specifically addressed in the erenumab trials. Migraine and symptoms of anxiety or depression are linked in a bidirectional fashion [28]; in the present case series, the improvement of psychiatric symptoms might be explained by the reduction in recurrent disabling migraine attacks which do not respond to treatment.

Our data also showed a reduction of allodynia symptoms, indicating a possible role of erenumab in the reversal of the sensitization to head pain typical of CM. Allodynia is a marker of central sensitization and is typical of CM [29]. Animal studies suggest that CGRP is implied in generating and maintaining allodynia [30, 31]; therefore, it is not surprising that allodynia symptoms might have been reversed by erenumab in patients with migraine. Notably, the prevalence of allodynia in our population was lower than in previous reports [32], suggesting potential underreporting or a mild effect of previous treatments.

We did not use detoxication for patients with medication overuse. A recent randomized controlled trial showed that detoxification alone is effective to improve migraine frequency and may avoid costly medications [33]. However, as already showed in a subgroup analysis of a randomized controlled trial [34], our data suggest that erenumab alone might help detoxifying patients with medication overuse.

Another important finding of our study was that a relevant proportion of patients with CM who had failed treatment with botulinum toxin A responded to erenumab. This finding points out that the mechanisms of action of botulinum toxin A and of erenumab are different, with two main consequences. First, erenumab might be offered to patients refractory to botulinum toxin. Second, botulinum toxin A and erenumab might be combined in the future, even at the expense of a high cost, to offer the best possible treatment to patients with severe migraine.

As erenumab is an expensive treatment, factors which predict late response should be identified. In our study, patients not responding to treatment had marginally significant higher median MMDs at baseline and a significantly higher consumption of analgesics compared with responders. We did not systematically assess the response to triptans in our patients.

The response to erenumab, as well as to any migraine treatment, is conditioned by the extreme variability of frequency and severity of migraine itself and cannot be univocally defined. A large proportion of patients not achieving a ≥ 50% response might still have a significant gain in terms of disability, associated symptoms, and drug consumption. Better tools are needed to assess the real improvement of patients with migraine after a treatment.

A further point of debate is the role of erenumab dose escalation from 70 mg to 140 mg monthly. In our observational study, dose escalation was allowed throughout the study period, and 2 patients started treatment with a 140 mg monthly dose. As we performed dose escalation at variable time intervals throughout the study, depending on the patients’ response, we could not assess the exact contribution of dose escalation to the efficacy and safety outcomes. Higher doses of erenumab might have given a substantial contribution to patients with a higher number of baseline MMDs (Table 2) or with medication overuse (Fig. 4).

We found comparable rates of adverse events, and especially serious adverse events, in our study as compared with the available randomized controlled trials (Table 4). The proportion of patients with constipation was higher in our study (13.5%) compared with the trials (0–3.6%) [17, 18, 26] and even open-label extensions [35, 36], possibly because patients and their treating physicians expected that event; however, it was mild and well controlled with diet or fibers in all cases and did not lead to discontinuation. Treatment discontinuation in our study was higher than in the available trials [17–19, 26] and mostly due to patients’ preference of discontinuing an ineffective treatment; only one patient discontinued the treatment due to an adverse event. Notably, we did not assess the prevalence of possible anti-drug antibodies; however, the current guidelines for the use of anti-CGRP antibodies do not indicate the routine measurement of anti-drug antibodies [14]. Overall, the great majority (85.4%) of patients were compliant to the treatment throughout the study period, further supporting the safety and tolerability of erenumab in clinical practice.

The strengths of the present study include its relatively large number of patients as compared with previous real-life studies [21, 22] and a remarkably longer follow-up of 6 months instead of two. The relatively large number of included patients allowed subgroup efficacy analyses in subgroup of patients with medication overuse and failure of botulinum toxin A. However, our study also has several limitations. Firstly, we could not assess the effect of concurrent oral preventive treatments, their withdrawal and reintroduction, due to heterogeneity and small numbers; however, this is a potential source of bias common to all real-life studies, in which treatments are prescribed on a case-by-case basis according to clinical needs; besides, such treatments were withdrawn or introduced in a minority of patients. Secondly, we did not assess some characteristics potentially linked to erenumab response, including prior response to triptans. Thirdly, the design of our study did not allow us to establish a definite role of dose escalation, as this procedure was decided during different time points according to patient response.

## Conclusion

Our real-life multicenter study showed the efficacy and safety of erenumab in a difficult-to-treat population of patients, most of whom had CM. The efficacy results were generally higher than those of the trials. The efficacy of erenumab was also shown in patients with medication overuse and in patients with CM and prior failure of treatment with botulinum toxin A. Further studies are needed to identify potential predictors of late response that might justify prolonged treatment and to provide real-life experience on extended treatment duration.

## Data Availability

Anonymized data operated or analyzed during this study are available from the Authors upon reasonable request.

## Abbreviations

AIFA: Italian Agency for Drug administration
ASC-12: Allodynia Symptom Checklist-12
BDI: Beck Depression Inventory
CGRP: Calcitonin gene-related peptide
CGRPr: Calcitonin gene-related peptide receptor
CM: Chronic migraine
EHF/LTB: European Headache Federation/ Lifting The Burden
EM: Episodic migraine
GAD-7: Generalized Anxiety Disorder Questionnaire
HIT-6: Headache Impact Test, 6th Edition
ICHD: International Classification of Headache Disorders
IQR: Interquartile range
LIBERTY: A 12-week Double-blind, Randomized, Multicenter Study Comparing the Efficacy and Safety of Once Monthly Subcutaneous AMG 334 Against Placebo in Adult Episodic Migraine Patients Who Have Failed Prophylactic Migraine Treatments
MIDAS: Migraine Impact and Disability Assessment Scale
MMDs: Monthly migraine days
NRS: Numerical Rating Scale
SD: Standard deviation
WHO: World Health Organization
